# Mothers with hypertensive disorders of pregnancy increased risk of periventricular leukomalacia in extremely preterm or extremely low birth weight infants: A propensity score analysis

**DOI:** 10.3389/fped.2022.978373

**Published:** 2022-08-23

**Authors:** Zhiwen Su, Weiliang Huang, Qiong Meng, Chunhong Jia, Bijun Shi, Xi Fan, Qiliang Cui, Jingsi Chen, Fan Wu

**Affiliations:** ^1^Department of Pediatrics, The Third Affiliated Hospital of Guangzhou Medical University, Guangzhou, China; ^2^Guangdong Provincial Key Laboratory of Major Obstetric Diseases, The Third Affiliated Hospital of Guangzhou Medical University, Guangzhou, China; ^3^Department of Pediatrics, Guangdong Second Provincial General Hospital, Guangzhou, China; ^4^Department of Obstetrics and Gynecology, Center for Reproductive Medicine/Department of Fetal Medicine and Prenatal Diagnosis/BioResource Research Center, The Third Affiliated Hospital of Guangzhou Medical University, Guangzhou, China

**Keywords:** hypertensive disorders of pregnancy, extremely preterm infant, extremely low birth weight infant, outcome, propensity score matching

## Abstract

**Background:**

At present, the conclusions about the impact of hypertensive disorders of pregnancy (HDP) on the clinical outcomes of preterm infants are inconsistent. This study used the propensity score matching (PSM) analysis to evaluate the effect of HDP on clinical outcomes of extremely preterm or extremely low birth weight (EP/ELBW) infants.

**Methods:**

Retrospective analysis was performed on the EP/ELBW infants discharged from 26 tertiary neonatal intensive care units or died during hospitalization from 2008 to 2017, who were divided into HDP group and non-HDP group. The six covariates including sex, gestational age, birth weight, twin or multiple pregnancy, antenatal steroids administration, and conception method were matched through the PSM method at a ratio of 1:1. The survival rate at discharge and the major clinical complications were compared between the two groups.

**Results:**

After matching the six covariates, compared with the non-HDP group, there was no significant difference in the survival rate at discharge (64 vs. 63.2%, *p* > 0.05), the incidence of bronchopulmonary dysplasia (BPD) or moderate to severe BPD in the HDP group (58.3 vs. 54.9%, *p* > 0.05; 5.2 vs. 6.2%, *p* > 0.05). The incidence of periventricular leukomalacia (PVL) in the HDP group was significantly increased (5.7 vs. 1.9%, *p* < 0.05).

**Conclusions:**

HDP increased the risk of PVL in EP/ELBW infants, but had no significant effect on the survival rate at discharge, or the occurrence of other complications.

## Introduction

Hypertensive disorders of pregnancy (HDP) are a major comorbidity or complication of pregnancy, accounting for about 10% of all pregnancies worldwide, and about 20% of preterm births are caused by HDP (1–3). HDP is characterized by hypertension in pregnancy including gestational hypertension, preeclampsia-eclampsia, chronic hypertension, and preeclampsia superimposed on chronic hypertension. Preeclampsia is defined as new onset of hypertension with either proteinuria or other maternal organ dysfunction after 20 weeks of gestation (1). If HDP is not identified and controlled, it can lead to adverse maternal and neonatal outcomes, including increased risk of maternal stroke, stillborn, preterm birth, and lower birth weight (1, 2).

Over the past years, several studies have reported the effect of HDP on the clinical outcomes of preterm infants, but these conclusions are contradictory, especially in mortality (4–8), bronchopulmonary dysplasia (BPD) (4, 5, 9–12), and intraventricular hemorrhage (IVH) (4, 5, 10, 13–15). The inconsistency of these results may be related to different study populations and inconsistent baseline characteristics of the subjects. Recently, a retrospective study was performed in Japan, a total of 21,659 extremely or very preterm infants were divided into HDP and non-HDP groups, at a ratio of 1:1 after stratification by four factors including gestational age, maternal age, year of delivery, and parity (13). The results showed that the mortality and the incidence of severe IVH were lower in the HDP group. Another study concluded that the factors such as sex, gestational age, birth weight, multiple births, and antenatal steroids could affect the mortality of extremely preterm (EP) infants (16), while the Japanese study only matched gestational age as an important factor, which might affect the conclusion. Hence, the aim of our study was to further evaluate the effects of HDP on clinical outcomes of EP or extremely low birth weight (ELBW) infants by using the propensity score matching (PSM) method to match the covariates including sex, gestational age, birth weight, twin or multiple pregnancy, antenatal steroids administration, and conception method.

## Materials and methods

### Participating centers

This study was a secondary analysis of the EP/ELBW infants' data of a multi-center clinical research collaborative group (17, 18). The research objects were from 26 tertiary neonatal intensive care units (NICUs) in Guangdong Province. The Third Affiliated Hospital of Guangzhou Medical University was responsible for coordinating this survey, where all the data were aggregated, stored, and analyzed. Each participating unit was responsible for the collection of case data in its hospital and was responsible for its authenticity and completeness. This study was approved by the Ethics Committee of the Third Affiliated Hospital of Guangzhou Medical University.

### Subjects and data collection

The population included in this study needed to meet all the following creteria: (1) gestational age below 28 weeks or birth weight <1000 grams; (2) admission to one of the NICUs of the collaborative hospitals within 24 h after birth; (3) discharged alive or died during hospitalization from January 1, 2008, to December 31, 2017. Infants with one of the following conditions were excluded: (1) the infants with various severe congenital malformations such as hereditary metabolic diseases, central nervous system malformations, and cardiovascular malformations; (2) the mothers with maternal comorbidities or complications in pregnancy, including diabetes, thyroid dysfunction, placental abruption, placenta previa, cervical incompetence, premature rupture of membranes, symptomatic infection before delivery, and other severe internal and external diseases, but not including HDP; (3) incomplete necessary information. Based on the mother with or without HDP, all the involved infants were divided into HDP group or non-HDP group. The neonatal and maternal clinical data were analyzed. The neonatal data included sex, gestational age, birth weight, small for gestational age (SGA), 1-min and 5-min Apgar score, pulmonary surfactant administration, mechanical ventilation, major complications, and survival rate at discharged. The maternal data included age, conception method, twin or multiple pregnancy, delivery mode, antenatal steroids administration, intrauterine fetal distress, and HDP.

### Definitions and classifications

According to the guidelines for the diagnosis and treatment of HDP (19), HDP was defined as systolic blood pressure ≥140 mmHg (1 mmHg = 0.133 kPa) and/or diastolic blood pressure ≥90 mmHg measured at least twice in the same arm. Neonatal respiratory distress syndrome (RDS) was diagnosed in preterm infants with respiratory distress shortly after birth and/or a compatible chest X-ray appearance (20). BPD was defined as oxygen dependency for at least 28 days, and the severity classifications were assessed at 36 weeks postmenstrual age or at discharge (21). The diagnosis and grading of necrotizing enterocolitis (NEC) were defined according to the modified Bell criteria (22). Retinopathy of prematurity (ROP) and its grades were defined by the international classification of ROP (23). The therapy for ROP included laser coagulation, intravitreal antivascular endothelial growth factor, and surgical treatment. Both IVH and periventricular leukomalacia (PVL) were diagnosed by cranial ultrasonography or magnetic resonance imaging (MRI). The Papile criterion was used to grade IVH, and grade III-IV was referred to severe IVH (24). PVL was defined as degeneration of white matter adjacent to the cerebral ventricles following cerebral hypoxia or brain ischemia (25). Because the diagnostic criteria of hospital acquired infection referred to the infections that occurred after 48 h of hospitalization (26), onset sepsis in this study was defined by clinical symptoms and positive culture from blood or cerebrospinal fluid samples after 48 h of admission. SGA was defined as sex-specific birth weight below the 10th percentile for gestational age (27). Antenatal steroid administration was defined as the use of dexamethasone or betamethasone to accelerate fetal lung maturity within 7 days before delivery (28). Fetal distress was defined as a syndrome that endangered the health and life of the fetus *in utero* due to acute or chronic hypoxia (28). Hemodynamically significant patent ductus arteriosus (hsPDA) was diagnosed by echocardiography and was defined as an arterial duct diameter>1.5 mm with diastolic flow reversal in the descending aorta, and a left atrial to aortic root rate >1.4 (29).

The main outcomes in this study were survival rate at discharged, and the occurrence of the complications including BPD, RDS, NEC, ROP, IVH, PVL, sepsis and hsPDA.

### Statistical analysis

All data were analyzed using SPSS 26.0 software (IBM, Armonk, NY, USA). We initiated a 1:1 matched analysis by PSM with a nearest-neighbor matching algorithm to adjust the baseline characteristic differences between the two groups, including sex, gestational age, birth weight, antenatal steroids administration, twin or multiple pregnancy, and conception through *in vitro* fertilization and embryo transfer (IVF-ET). These covariates were selected based on the previous studies, which were found to be relative to the outcomes of preterm infants (16, 30–32). We used calipers of width equal to 0.02 of the standard deviation of the logit of the propensity score. The normality of the data distribution was shown as means ± standard deviation (SD), which was analyzed using *t-*test. Categorical variables were presented as rates, which were analyzed using *Chi-square* tests. When *P* < 0.05 is considered as significant difference.

## Results

### Baseline characteristics of neonates and mothers

**Figure 1:**
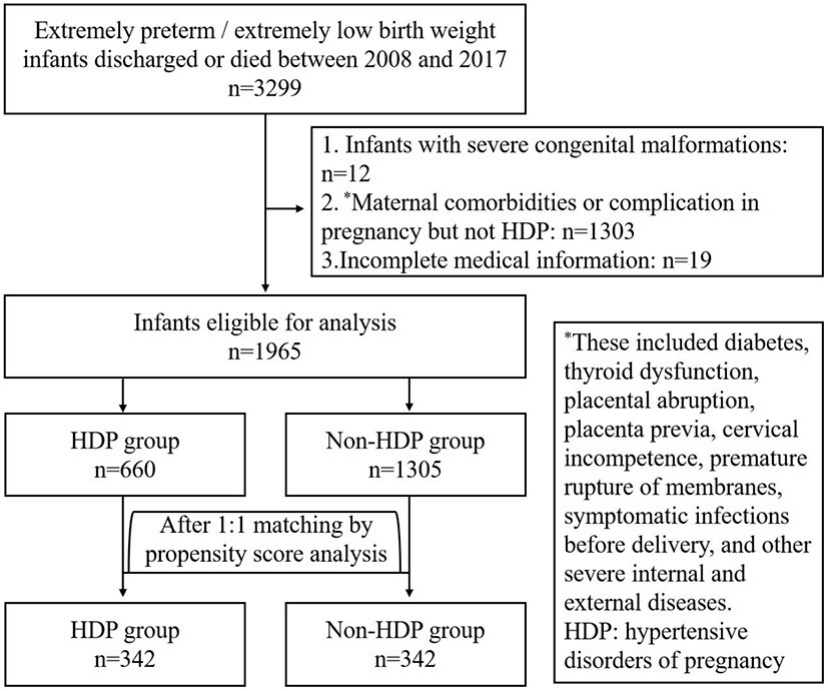
Flow chart of enrolment of the study population.

Between 2008 and 2017, there were 3,299 EP/ELBW infants discharged from the included hospitals or died during the hospitalization. After exclusion of 1,334 cases, the rest 1,965 cases of EP/ELBW infants were enrolled, and they were divided into the HDP group and the non-HDP group (Figure 1). Before matching, there were 660 infants in the HDP group and 1,305 infants in the non-HDP group. After matching, each group included 342 infants. The baseline characteristics of the infants and mothers in the two groups before and after matching are shown in Tables 1, 2 respectively. Before matching, 12 of the 14 covariables including male, gestational age, birth weight, SGA, surfactant therapy, mechanical ventilation of the infants; and elderly maternal age (≥35 years), IVF-ET, antenatal steroids administration, twin or multiple pregnancy, intrauterine fetal distress, cesarean section, were significantly different between the two groups. After matching, only elderly maternal age (≥35 years), cesarean section and 1-min Apgar score were significantly different between the two groups.

**Table 1:** The neonatal characteristics in HDP and non-HDP groups.

	**Total (*N* = 1,965)**	**Before matching**			**After matching**		
		**HDP** **(*N* = 660)**	**Non-HDP** **(*N =* 1,305)**	***P*-value**	**HDP** **(*N =* 342)**	**Non-HDP** **(*N =* 342)**	***P-*value**
Male sex, *N* (%)	1,131 (57.6)	342 (51.8)	789 (60.5)	<0.001	191 (55.8)	182 (53.2)	0.490
Gestational age (weeks), mean±SD	27.94 ± 1.94	29.10 ± 1.80	27.35 ± 1.73	<0.001	28.42 ± 1.63	28.43 ± 2.23	0.910
<27 weeks, *N* (%)	519 (26.4)	59 (8.9)	460 (35.2)		52 (15.2)	70 (19.9)	
27-28 weeks, *N* (%)	915 (46.6)	256 (38.8)	659 (50.5)	<0.001	171 (50.0)	156 (44.3)	0.183
≥29 weeks, *N* (%)	531 (27.0)	345 (52.3)	186 (14.3)		119 (34.8)	126 (35.8)	
Birth weight (grams), mean±SD	911 ± 158	869 ± 132	932 ± 166	<0.001	892 ± 135	893 ± 138	0.949
SGA, *N* (%)	446 (22.7)	294 (44.5)	152 (11.6)	<0.001	97 (28.4)	110 (32.2)	0.279
Apgar ≤ 7 at 1 min, *N* (%)	927 (47.2)	318 (48.2)	609 (46.7)	0.525	166 (48.5)	133 (38.9)	0.011
Apgar ≤ 7 at 5 min, *N* (%)	344 (17.5)	105 (15.9)	239 (18.3)	0.185	53 (15.5)	51 (14.9)	0.831
Pulmonary surfactant administration, *N* (%)	1,255 (63.9)	390 (59.1)	865 (66.3)	0.002	268 (78.4)	256 (74.9)	0.278
Mechanical ventilation, *N* (%)	1,347 (68.5)	429 (65.0)	918 (70.3)	0.016	237 (69.3)	224 (65.5)	0.289

*EP, extremely preterm; ELBW, extremely low birth weight; HDP, hypertensive disorders of pregnancy; SD, standard deviation; SGA, small for gestational age*.

**Table 2:** The maternal characteristics in HDP and non-HDP groups.

	**Total (*N =* 1,965)**	**Before matching**			**After matching**		
		**HDP (*N =* 660)**	**Non-HDP (*N =* 1,305)**	***P*-value**	**HDP (*N =* 342)**	**Non-HDP (*N =* 342)**	***P-*value**
Maternal age ≥35 years, *N* (%)	388 (19.7)	208 (31.5)	180 (13.8)	<0.001	111 (32.5)	53 (15.5)	<0.001
IVF-ET, *N* (%)	246 (12.5)	55 (8.3)	180 (13.8)	<0.001	28 (8.2)	28 (8.2)	>0.999
Twin or multiple pregnancy, *N* (%)	669 (34.0)	121 (18.3)	548 (42.0)	<0.001	83 (24.3)	88 (25.7)	0.659
Antenatal steroids, *N* (%)	918 (46.7)	358 (54.2)	191 (14.6)	<0.001	215 (62.9)	220 (64.3)	0.691
Cesarean section, *N* (%)	794 (40.4)	572 (86.7)	222 (17.0)	<0.001	291 (85.1)	103 (30.1)	<0.001
Intrauterine fetal distress, *N* (%)	124 (6.3)	82 (12.4)	42 (3.2)	<0.001	39 (11.4)	27 (7.9)	0.120

*IVF-ET, in vitro fertilization and embryo transfer; HDP, hypertensive disorders of pregnancy*.

### Clinical outcomes

Before matching, the survival rate at discharge of the HDP group was significantly higher than that of the non-HDP group (62.7% [414/660] vs. 53.1% [693/1,305], *P* < 0.01). After matching, there was no significant difference in it between the two groups.

On the other hand, compared with the non-HDP group, the incidences of total BPD, ROP above stage II or required therapy, total IVH or severe IVH (grade III-IV), and hsPDA in the HDP group were lower before matching (all *P* < 0.01), but they had no significant difference after matching. Oppositely, before matching, the HDP group had a significantly higher incidence of sepsis occurred after 48 h admission (*P* < 0.01), but not after matching. The incidence of PVL between the two groups was not significantly different before matching (6.2% [30/486] vs. 5.3% [51/961], *P* > 0.05), while it was higher in the HDP group (5.7% [14/246] vs. 1.9% [5/266], *P* < 0.05) after matching. In addition, there was no significant difference in the incidence of RDS, moderate to severe BPD, total ROP, total NEC or definite NEC (≥stage IIa) between the two groups before or after matching. The results were shown in Table 3.

**Table 3:** The clinical outcomes of EP/ELBW infants in HDP and non-HDP groups.

	**Total (*N =* 1,965)**	**Before matching**			**After matching**		
		**HDP** **(*N =* 660)**	**Non-HDP** **(*N =* 1,305)**	***P*-value**	**HDP (*N =* 342)**	**Non-HDP** ***(N = 342)***	***P*-value**
Major outcome, *N* (%)							
Survived at discharge	1,107 (56.3)	414 (62.7)	693 (53.1)	<0.001	219 (64.0)	216 (63.2)	0.812
Complications, diagnosed *N*/assessed *N* (%)							
RDS ^a^	1,657/1,965 (84.3)	553/660 (83.8)	1,104/1,305 (84.6)	0.641	301/342 (88.0)	283/342 (82.7)	0.051
BPD (total) ^b^	699/1,182 (59.1)	226/441 (51.2)	473/741 (63.8)	<0.001	134/230 (58.3)	124/226 (54.9)	0.465
Moderate to severe BPD ^b^	66/1,182 (5.6)	19/441 (4.3)	47/741 (6.3)	0.141	12/230 (5.2)	14/226 (6.2)	0.653
NEC (total) ^c^	201/1,542 (13.0)	80/556 (14.4)	121/986 (12.3)	0.236	35/288 (12.2)	38/284 (13.4)	0.660
Definite NEC (≥ stage IIa) ^c^	85/1,542 (5.5)	34/556 (6.1)	51/986 (5.2)	0.436	14/288 (4.9)	17/284 (6.0)	0.552
ROP (total) ^d^	447/1,122 (39.8)	151/417 (36.2)	296/705 (42.0)	0.056	88/216 (40.7)	79/217 (36.4)	0.354
ROP (≥ stage II) ^d^	274/1,122 (24.4)	71/417 (17.0)	203/705 (28.8)	<0.001	42/216 (19.4)	49/217 (22.6)	0.423
ROP required therapy ^d^	66/1,122 (5.9)	16/417 (3.8)	50/705 (7.1)	0.025	14/216 (6.5)	13/217 (6.0)	0.833
IVH (total) ^e^	428/1,447 (29.6)	112/486 (23.0)	316/961 (32.9)	<0.001	54/246 (22.0)	70/266 (26.3)	0.249
Severe IVH (grade III-IV) ^e^	118/1,447 (8.2)	20/486 (4.1)	98/961 (10.2)	<0.001	9/246 (3.7)	14/266 (5.3)	0.381
PVL ^e^	81/1,447 (5.6)	30/486 (6.2)	51/961 (5.3)	0.499	14/246 (5.7)	5/266 (1.9)	0.023
Sepsis ^f^	249/1,542 (16.1)	110/556 (19.8)	139/986 (14.1)	0.004	61/288 (21.2)	43/284 (15.1)	0.061
hsPDA ^g^	444/1,465 (30.3)	127/509 (25.0)	317/956 (33.2)	0.001	69/282 (24.5)	58/298 (19.5)	0.145

*EP, extremely preterm; ELBW, extremely low birth weight; HDP, hypertensive disorders of pregnancy; RDS, respiratory distress syndrome; BPD, bronchopulmonary dysplasia; NEC, necrotizing enterocolitis; ROP, retinopathy of prematurity; IVH, intraventricular hemorrhage; PVL, periventricular leukomalacia; hsPDA, hemodynamically significant patent ductus arteriosus*.

a *RDS was diagnosed in preterm infants with respiratory distress shortly after birth and/or a compatible chest X-ray appearance, all the enrolled infants were assessed*.

b *BPD was defined as oxygen dependency for at least 28 days, and the infants survived more than 28 days were assessed. The severity classifications were assessed at 36 weeks postmenstrual age or at discharge*.

c *NEC was diagnosed and graded according to modified Bell criteria, and the infants survived more than 48 h were assessed*.

d *ROP was assessed in the infants underwent direct ophthalmoscopy or ret-camera examination, and graded by the international classification of ROP. ROP required therapy referred to the individuals who received treatment for ROP such as laser coagulation, intravitreal antivascular endothelial growth factor, or surgical treatment*.

e *IVH and PVL was assessed in the infants underwent cranial ultrasonography or magnetic resonance imaging (MRI) examination. The Papile criterion was used to grade IVH, and grade III-IV was defined as severe IVH. PVL was defined as degeneration of white matter adjacent to the cerebral ventricles following cerebral hypoxia or brain ischemia*.

f *Sepsis was defined by clinical symptoms and positive culture from blood or cerebrospinal fluid samples after 48 h of admission. The infants survived more than 48 h were assessed*.

g *hsPDA was defined as an arterial duct diameter >1.5 mm with diastolic flow reversal in the descending aorta, and a left atrial to aortic root rate >1.4. The infants underwent cardiac ultrasonography examination were assessed*.

## Discussion

In our study, after matching the six major covariates including sex, gestational age, birth weight, antenatal steroids administration, conception through IVF-ET, and twin or multiple pregnancy, mothers with HDP increased the risk of PVL in EP/ELBW infants, but had no significant effect on the survival rate at discharge, or the incidence of other complications.

After marching, survival was not significantly different in the two groups. Our data showed that the HDP group had a significant higher survival rate at discharge before matching, which was similar to the report of Gemmell et al. (4–6). This may be related to the following aspects: firstly, the delivery method of pregnant women with HDP is usually planned, and it can help improve antenatal steroids administration and reduce the mortality of EP infants (33, 34). Secondly, in the present study, compared with the non-HDP group, the proportion of SGA infants in the HDP group was significantly higher before matching, while it was reported that SGA infants of HDP mothers had a lower mortality rate (10). Finally, as a great contributor to overall risk in preterm infants (35), the gestational age was larger in HDP group before matching. Another study found that the factors such as sex, gestational age, birth weight, multiple births, and antenatal steroids could affect the mortality of EP infants (16). In our study, after matching these factors, there was no significant difference in survival rate between the two groups, which was consistent with the results of Kono et al. (7, 8), but inconsistent with the results reported in Japan (13).

HDP had little effect on the risk of BPD in EP/ELBW infants. To date, the results of researches on the impact of HDP on the development of BPD are not uniform. Some studies discovered that HDP could increase the risk for BPD (4, 5, 9), but some reports supported the opposite view (10–12), and some others found no difference (36–38). Like the results of the study by ElSayed et al. (10, 12), our study showed that the HDP group had a lower incidence of BPD before matching. This may be related to the following: firstly, gestational age is the strongest predictive factor for BPD. The infant with a smaller gestational age has a higher risk of BPD (39). In our study, the gestational age of the HDP group was greater than that of the non-HDP group before matching. Secondly, ElSayed et al. (10) demonstrated that SGA infants of HDP mothers had a lower incidence of BPD than those of non-HDP mothers. In our study, the HDP group had a higher proportion of SGA infants before matching. After matching for the relevant factors, this study showed that there was no significant difference in the incidence of BPD or moderate to severe BPD between the two groups. This was consistent with the conclusions of O'Shea et al. (36–38). O'Shea et al. (36) found that there was no significant difference in the incidence of BPD in EP/ELBW infants in the preeclampsia group compared with the non-preeclampsia group. After adjusting for confounding variables such as sex, gestational age, and birth weight, BPD was not associated with preeclampsia. Another retrospective study included 1,827 preterm infants with gestational age below 30 weeks in the Korean Neonatal Network, and the infants were divided into pregnancy-induced hypertension (PIH) groups and non-PIH groups. It showed that the incidence of BPD was not significantly different between the two groups; after adjusting for sex, gestational age, RDS, and treated PDA, there was no correlation between PIH and BPD in EP infants below 27 weeks of gestation (37). As the risk factors for BPD are multifactorial, in addition to abnormal vascular growth caused by maternal factors, there are many other risk factors that can contribute to the development of BPD (37), while gestational age and low birth weight are the two strongest risk factors (39). Therefore, after matching gestational age, birth weight and sex, HDP had no significant effect on the incidence of BPD in EP/ELBW infants.

HDP increased the risk of PVL in EP/ELBW infants. The IVH and PVL are the two major causes leading to neurological sequelae of preterm infants. The reported studies demonstrated that the correlation between HDP and IVH/PVL was also contradictory (4, 5, 10, 13–15). In this study, before matching, EP/ELBW infants in the HDP group had a significantly lower incidence of IVH or severe IVH. This was similar to the results of Gemmell et al. (4, 5, 14, 15). After matching, there was no significant difference in the incidence of IVH or severe IVH in EP/ELBW infants between the HDP group and the non-HDP group. This was consistent with the results reported by ElSayed et al. (10), but it was contrary to the recent report of Nakamura et al. (13). The study conducted by Nakamura et al. also matched at the rate of 1:1, but the matching factors were different from our study. The two major factors including birth weight and antenatal steroids were not matched. The administration of antenatal steroids could significantly reduce the risk of IVH in preterm infants (40), as well as the incidence of IVH in preterm infants with HDP (41). In this study, the proportion of antenatal steroids administration in the HDP group was significantly higher than that of non-HDP before matching; but after matching, the proportions of antenatal steroids administration in the two groups were similar. This could explain why there was no difference in the incidence of IVH between the two groups after matching in our study. It also suggests that HDP has little effect on the occurrence of IVH in EP/ELBW infants, which is consistent with the previous view that “preeclampsia has no significant influence on the occurrence of IVH” (42). In addition, our results also found that after matching, the incidence of PVL in the HDP group was significantly higher than that in the non-HDP group, which was inconsistent with the results reported by Nakamura et al. (13). This shows that HDP has an impact on the occurrence of PVL under the premise that gestational age, birth weight, sex and antenatal steroids administration are consistent. It may be related to the pathogenesis of PVL. Hypoxia is one of the two major upstream mechanisms of PVL (43). In HDP mothers, especially preeclampsia, the fetus is exposed to chronic hypoxia in the intrauterine environment (44). Placental hypoxia leads to oligodendrocyte dysmaturation in the offspring, resulting in impaired myelination (45), and ultimately forming PVL. At the same time, the animal model of preeclampsia also found that preeclampsia could induce oligodendrocytes death and demyelination in the offspring (46). Studies noted that PVL was associated with Autism Spectrum Disorders (ASD) (47), and HDP could increase the risk of ASD (48, 49). Does it suggest that there are any associations among HDP, PVL and ASD? The further research is needed. In this study, compared with the non-HDP group, the proportion of 1-min Apgar scores <7 was significantly higher in the HDP group after matching. A systematic review and meta-analysis showed that infants with 1-min Apgar score at birth <7 had an increased risk of PVL (50). This might also be one of the risk factors for the increased incidence of PVL in the HDP group in our study.

In addition, our results showed that the incidence of onset sepsis after 48 h admission was significantly higher in the HDP group before matching. This is similar to the study of Letouzey et al. (51), which found that very preterm infants with HDP had a higher risk of late-onset sepsis. The causes for it might be premature infants with HDP susceptible to neutropenia or low plasma immunoglobulin concentration (51). The inflammatory response induced by infection is also an important mechanism leading to the occurrence of PVL.

In summary, HDP increased the risk of PVL in EP/ELBW infants, but has no significant effect on the survival rate at discharged, or the occurrence of other complications. However, some limits remain in this study. First, it was a secondary analysis by PSM method, not a randomized controlled study. Second, there may be some other factors such as histological chorioamnionitis which lacked placental histological data in this study that affect the clinical outcomes of EP/ELBW infants, as well as the six matched covariates. Third, due to the lack of data on pulmonary hypertension, we couldn't analyze the association between the presence of HDP and the pulmonary hypertension of the infants, especially the vascular phenotype associated with BPD. Thus, the further studies are needed.

## Data availability statement

The raw data supporting the conclusions of this article will be made available by the authors, without undue reservation.

## Ethics statement

This study was approved by the Medical Ethics Committee of the Third Affiliated Hospital of Guangzhou Medical University.

## Author contributions

FW, QM, and JC: study concept and design. ZS, WH, and XF: drafting of the manuscript. FW, QC, and BS: statistical analyses. FW, CJ, and ZS: review and editing. JC and FW: funding acquisition. All authors contributed to the article and approved the submitted version.

## Funding

This work was supported by the Natural Science Foundation of Guangdong Province (No. 2020A1515010273 to JC) and Guangzhou Science and Technology Project (No. 202102010080 to FW).

## Conflict of interest

The authors declare that the research was conducted in the absence of any commercial or financial relationships that could be construed as a potential conflict of interest.

## Publisher's note

All claims expressed in this article are solely those of the authors and do not necessarily represent those of their affiliated organizations, or those of the publisher, the editors and the reviewers. Any product that may be evaluated in this article, or claim that may be made by its manufacturer, is not guaranteed or endorsed by the publisher.

## References

[1] Webster K Fishburn S Maresh M Findlay SC Chappell LC Guideline C. Diagnosis and management of hypertension in pregnancy: summary of updated nice guidance. BMJ. (2019) 366:l5119. 10.1136/bmj.l5119 31501137

[2] American College of Obstetricians and Gynecologists. ACOG Practice Bulletin No. 202: Gestational Hypertension and Preeclampsia. Obstet Gynecol. (2019) 133:e1-25. 10.1097/AOG.0000000000003018 30575675

[3] Roberts CL Algert CS Morris JM Ford JB Henderson-Smart DJ. Hypertensive disorders in pregnancy: a population-based study. Med J Aust. (2005) 182:332–5. 10.5694/j.1326-5377.2005.tb06730.x 15804223

[4] Gemmell L Martin L Murphy KE Modi N Hakansson S Reichman B. Hypertensive disorders of pregnancy and outcomes of preterm infants of 24 to 28 weeks' gestation. J Perinatol. (2016) 36:1067–72. 10.1038/jp.2016.133 27583388

[5] Razak A Florendo-Chin A Banfield L Abdul Wahab MG McDonald S Shah PS. Pregnancy-induced hypertension and neonatal outcomes: a systematic review and meta-analysis. J Perinatol. (2018) 38:46–53. 10.1038/jp.2017.162 29095432

[6] Gagliardi L Basso O. Maternal hypertension and survival in singletons and twins born at 23-29 weeks: not just one answer. Pediatr Res. (2019) 85:697–702. 10.1038/s41390-019-0337-4 30763949

[7] Kono Y Yonemoto N Nakanishi H Hosono S Hirano S Kusuda S. A retrospective cohort study on mortality and neurodevelopmental outcomes of preterm very low birth weight infants born to mothers with hypertensive disorders of pregnancy. Am J Perinatol. (2021). 10.1055/s-0041-1722874 33535243

[8] Lu CQ Lin J Yuan L Zhou JG Liang K Zhong QH. Pregnancy induced hypertension and outcomes in early and moderate preterm infants. Pregnancy Hypertens. (2018) 14:68–71. 10.1016/j.preghy.2018.06.008 30527121

[9] Tagliaferro T Jain D Vanbuskirk S Bancalari E Claure N. Maternal preeclampsia and respiratory outcomes in extremely premature infants. Pediatr Res. (2019) 85:693–6. 10.1038/s41390-019-0336-5 30770862

[10] ElSayed E Daspal S Yee W Pelausa E Canning R Shah PS. Outcomes of singleton small for gestational age preterm infants exposed to maternal hypertension: a retrospective cohort study. Pediatr Res. (2019) 86:269–75. 10.1038/s41390-019-0416-6 31086284

[11] Yen TA Yang HI Hsieh WS Chou HC Chen CY Tsou KI. Preeclampsia and the risk of bronchopulmonary dysplasia in VLBW infants: a population based study. PLoS ONE. (2013) 8:e75168. 10.1371/journal.pone.0075168 24073247 PMC3779258

[12] Sloane AJ Flannery DD Lafferty M Jensen EA Dysart K Cook A. Hypertensive disorders during pregnancy are associated with reduced severe intraventricular hemorrhage in very-low-birth-weight infants. J Perinatol. (2019) 39:1125–30. 10.1038/s41372-019-0413-y 31263202

[13] Nakamura N Ushida T Nakatochi M Kobayashi Y Moriyama Y Imai K. Mortality and neurological outcomes in extremely and very preterm infants born to mothers with hypertensive disorders of pregnancy. Sci Rep. (2021) 11:1729. 10.1038/s41598-021-81292-7 33462302 PMC7814115

[14] Bossung V Fortmann MI Fusch C Rausch T Herting E Swoboda I. Neonatal outcome after preeclampsia and Hellp syndrome: a population-based cohort study in Germany. Front Pediatr. (2020) 8:579293. 10.3389/fped.2020.579293 33154958 PMC7586782

[15] Morsing E Marsal K Ley D. Reduced prevalence of severe intraventricular hemorrhage in very preterm infants delivered after maternal preeclampsia. Neonatology. (2018) 114:205–11. 10.1159/000489039 29940569

[16] Rysavy MA Horbar JD Bell EF Li L Greenberg LT Tyson JE. Assessment of an updated neonatal research network extremely preterm birth outcome model in the Vermont Oxford Network. JAMA Pediatr. (2020) 174:e196294. 10.1001/jamapediatrics.2019.6294 32119065 PMC7052789

[17] Collaborative Study Group for Extremely Preterm and Extremely Low Birth Weight Infants. [Survival and mortality rate of extremely preterm and extremely low birth weight infants admitted to neonatology departments]. Zhonghua Er Ke Za Zhi. (2014) 52:729–35. 10.3760/cma.j.issn.0578-1310.2014.10.003 25537536

[18] Collaborative Study Group for Extremely Preterm and Extremely Low Birth Weight Infants. [Short-term outcomes and their related risk factors of extremely preterm and extremely low birth weight infants in Guangdong Province]. Zhonghua Er Ke Za Zhi. (2019) 57:934-42. 10.3760/cma.j.issn.0578-1310.2019.12.008 31795560

[19] Magee LA Pels A Helewa M Rey E von Dadelszen P. Canadian Hypertensive Disorders of Pregnancy (HDP) working group. Diagnosis, evaluation, and management of the hypertensive disorders of pregnancy. Pregnancy Hypertens. (2014) 4:105–45. 10.1016/j.preghy.2014.01.003 26104418

[20] Bhakta K. Respiratory Distress Syndrome In: Manual of Neonatal Care, 7th edition. Philadelphia: Little, Brown and Company (2012). p. 406–16.

[21] Jobe AH Bancalari E. Bronchopulmonary dysplasia. Am J Respir Crit Care Med. (2001) 163:1723–9. 10.1164/ajrccm.163.7.2011060 11401896

[22] Walsh MC Kliegman RM. Necrotizing enterocolitis: treatment based on staging criteria. Pediatr Clin North Am. (1986) 33:179–201. 10.1016/S0031-3955(16)34975-6 3081865 PMC7131118

[23] International Committee for the Classification of Retinopathy of Prematurity. The international classification of retinopathy of prematurity revisited. Arch Ophthalmol. (2005) 123:991-9. 10.1001/archopht.123.7.991 16009843

[24] Papile LA Burstein J Burstein R Koffler H. Incidence and evolution of subependymal and intraventricular hemorrhage: a study of infants with birth weights less than 1,500 gm. J Pediatr. (1978) 92:529–34. 10.1016/S0022-3476(78)80282-0 305471

[25] Volpe JJ. Neurobiology of periventricular leukomalacia in the premature infant. Pediatr Res. (2001) 50:553–62. 10.1203/00006450-200111000-00003 11641446

[26] Ministry of Health of the People's Republic of China. Diagnostic criteria for nosocomial infections (proposed). Zhonghua yi xue za zhi. (2001) 81:314–20. 10.3760/j:issn:0376-2491.2001.05.027

[27] Boghossian NS Geraci M Edwards EM Horbar JD. Morbidity and mortality in small for gestational age infants at 22 to 29 weeks' gestation. Pediatrics. (2018) 141:e20172533. 10.1016/j.jpeds.2018.02.042 29348195

[28] Xie Xing KB DT. Obstetrics and Gynecology. 9th edition. China: People's Medical Publishing House (2018). 96–7p.

[29] Jain A Shah PS. Diagnosis, evaluation, and management of patent ductus arteriosus in preterm neonates. JAMA Pediatr. (2015) 169:863–72. 10.1001/jamapediatrics.2015.0987 26168357

[30] Berntsen S Soderstrom-Anttila V Wennerholm UB Laivuori H Loft A Oldereid NB. The health of children conceived by art: ‘the chicken or the egg?’ Hum Reprod Update. (2019) 25:137–58. 10.1093/humupd/dmz001 30753453

[31] Bader D Kugelman A Boyko V Levitzki O Lerner-Geva L Riskin A. Risk factors and estimation tool for death among extremely premature infants: a national study. Pediatrics. (2010) 125:696–703. 10.1542/peds.2009-1607 20351002

[32] Chawla S Natarajan G Shankaran S Pappas A Stoll BJ Carlo WA. Association of neurodevelopmental outcomes and neonatal morbidities of extremely premature infants with differential exposure to antenatal steroids. JAMA Pediatr. (2016) 170:1164–72. 10.1001/jamapediatrics.2016.1936 27723868 PMC5294968

[33] Carlo WA McDonald SA Fanaroff AA Vohr BR Stoll BJ Ehrenkranz RA. Association of antenatal corticosteroids with mortality and neurodevelopmental outcomes among infants born at 22 to 25 weeks' gestation. JAMA. (2011) 306:2348–58. 10.1001/jama.2011.1752 22147379 PMC3565238

[34] Mori R Kusuda S Fujimura M Neonatal Research Network J. Antenatal corticosteroids promote survival of extremely preterm infants born at 22 to 23 weeks of gestation. J Pediatr. (2011) 159:110-4 e1. 10.1016/j.jpeds.2010.12.039 21334006

[35] Gutbrod T Wolke D Soehne B Ohrt B Riegel K. Effects of gestation and birth weight on the growth and development of very low birthweight small for gestational age infants: a matched group comparison. Arch Dis Child Fetal Neonatal Ed. (2000) 82:F208–14. 10.1136/fn.82.3.F208 10794788 PMC1721075

[36] O'Shea JE Davis PG Doyle LW. Victorian Infant Collaborative Study Group. Maternal Preeclampsia and Risk of Bronchopulmonary Dysplasia in Preterm Infants Pediatr Res. (2012) 71:210–4. 10.1038/pr.2011.27 22258134

[37] Shin SH Shin SH Kim SH Kim YJ Cho H Kim EK. The association of pregnancy-induced hypertension with bronchopulmonary dysplasia - a retrospective study based on the Korean neonatal network database. sci Rep. (2020) 10:5600. 10.1038/s41598-020-62595-7 32221404 PMC7101434

[38] Pierro M Villamor-Martinez E van Westering-Kroon E Alvarez-Fuente M Abman SH Villamor E. Association of the dysfunctional placentation endotype of prematurity with bronchopulmonary dysplasia: a systematic review, meta-analysis and meta-regression. Thorax. (2022) 77:268–75. 10.1136/thoraxjnl-2020-216485 34301740 PMC8867288

[39] Thebaud B Goss KN Laughon M Whitsett JA Abman SH Steinhorn RH. Bronchopulmonary Dysplasia. Nat Rev Dis Primers. (2019) 5:78. 10.1038/s41572-019-0127-7 31727986 PMC6986462

[40] Shepherd E Salam RA Middleton P Makrides M McIntyre S Badawi N. Antenatal and intrapartum interventions for preventing cerebral palsy: an overview of cochrane systematic reviews. Cochrane Database Syst Rev. (2017) 8:CD012077. 10.1002/14651858.CD012077.pub2 28786098 PMC6483544

[41] Ushida T Kotani T Hayakawa M Hirakawa A Sadachi R Nakamura N. Antenatal corticosteroids and preterm offspring outcomes in hypertensive disorders of pregnancy: a Japanese cohort study. Sci Rep. (2020) 10:9312. 10.1038/s41598-020-66242-z 32518309 PMC7283214

[42] Bordbar A Farjadnia M. Maternal morbidities and occurrence of intraventricular hemorrhage in preterm infants. J Pediatr Intensive Care. (2015) 4:156–61. 10.1055/s-0035-1559825 31110865 PMC6513166

[43] Schneider J Miller SP. Preterm brain injury: white matter injury. Handb Clin Neurol. (2019) 162:155–72. 10.1016/B978-0-444-64029-1.00007-2 31324309

[44] Mol BWJ Roberts CT Thangaratinam S Magee LA de Groot CJM Hofmeyr GJ. Pre-Eclampsia. Lancet. (2016) 387:999–1011. 10.1016/S0140-6736(15)00070-7 26342729

[45] Gumusoglu SB Chilukuri ASS Santillan DA Santillan MK Stevens HE. Neurodevelopmental Outcomes of Prenatal Preeclampsia Exposure. Trends Neurosci. (2020) 43:253–68. 10.1016/j.tins.2020.02.003 32209456 PMC7170230

[46] Ijomone OK Shallie PD Naicker T. Oligodendrocytes death induced sensorimotor and cognitive deficit in N-nitro-L-arginine methyl rat model of pre-eclampsia. Neurochem Res. (2020) 45:902–14. 10.1007/s11064-020-02969-5 31983010

[47] Brossard-Racine M du Plessis AJ Limperopoulos C. Developmental cerebellar cognitive affective syndrome in ex-preterm survivors following cerebellar injury. Cerebellum. (2015) 14:151–64. 10.1007/s12311-014-0597-9 25241880 PMC4573589

[48] Maher GM O'Keeffe GW Kearney PM Kenny LC Dinan TG Mattsson M. Association of hypertensive disorders of pregnancy with risk of neurodevelopmental disorders in offspring: a systematic review and meta-analysis. JAMA Psychiatry. (2018) 75:809–19. 10.1001/jamapsychiatry.2018.0854 29874359 PMC6143097

[49] Brand JS Lawlor DA Larsson H Montgomery S. Association between hypertensive disorders of pregnancy and neurodevelopmental outcomes among offspring. JAMA Pediatr. (2021) 175:577–85. 10.1001/jamapediatrics.2020.6856 33749704 PMC7985818

[50] Huang J Zhang L Kang B Zhu T Li Y Zhao F. Association between perinatal hypoxic-ischemia and periventricular leukomalacia in preterm infants: a systematic review and meta-analysis. PLoS ONE. (2017) 12:e0184993. 10.1371/journal.pone.0184993 28931047 PMC5607162

[51] Letouzey M Foix-L'Helias L Torchin H Mitha A Morgan AS Zeitlin J. Cause of preterm birth and late-onset sepsis in very preterm infants: the epipage-2 cohort study. Pediatr Res. (2021) 90:584–92. 10.1038/s41390-021-01411-y 33627822 PMC7903216

